# Selection and characterization of probiotic lactic acid bacteria and its impact on growth, nutrient digestibility, health and antioxidant status in weaned piglets

**DOI:** 10.1371/journal.pone.0192978

**Published:** 2018-03-08

**Authors:** Runjun Dowarah, Ashok Kumar Verma, Neeta Agarwal, Putan Singh, Bhoj Raj Singh

**Affiliations:** 1 Centre of Advanced Faculty Training in Animal Nutrition, ICAR-Indian Veterinary Research Institute, Izatnagar, UP, India; 2 Division of Epidemiology, ICAR-Indian Veterinary Research Institute, Izatnagar, UP, India; Maharshi Dayanand University, INDIA

## Abstract

The present study was aimed to develop an effective probiotic lactic acid bacteria (LAB) from piglet feces and *in vitro* characterization of probiotic properties. To confirm host-species specificity of probiotics, the efficacy of isolated LAB on growth, nutrient utilization, health and antioxidant status was observed in early weaned piglets. A total of 30 LAB were isolated from feces of five healthy piglets (28d old). All isolates were Gram positive, cocco-bacilli and catalase negative. Out of thirty LAB isolates, twenty were shortlisted on the basis of their tolerance to pH (3.0, 4.0, 7.0 and 8.0) and bile salts (0.075, 0.15, 0.3 and 1.0%). Whereas, fourteen isolates were selected for further *in vitro* probiotic characterization due higher (P<0.05) cell surface hydrophobicity to toluene (>45 percent). These isolates fermented twenty-seven different carbohydrates but were negative for ONPG, citrate and malonate. Also enabled to synthesize amylase, protease, lipase and phytase. They were sensitive to penicillin, azithromycin, lincomycin, clindamycin, erythromycin, cephalothin and chloramphenicol and resistant to ciprofloxacin, ofloxacin, gatifloxacin, vancomycin and co-trimoxazole. Except three isolates, all showed antagonistic activity (>60% co-culture activity) against *Escherichia coli*, *Salmonella* Enteritidis, *Salmonella* serotype (ser.) Typhimurium, *Staphylococcus intermedius*, *Staph*. *chromogenes*, *Proteus mirabillis*, *Areomonas veonii*, *Bordetella bronchioseptica* and *Klebsialla oxytoca*. The isolate Lacp28 exhibited highest tolerance to acidic *p*H and bile salts (up to 0.3%), phytase activity, cell surface hydrophobicity, antagonistic activity and co-culture assay (>80% growth inhibition). Host specificity of Lacp28 was further confirmed by heavy *in vitro* adhesion to pig intestinal epithelium cells compared to chicken. Hence, Lacp28 was selected and identified by phylogenetic analysis of 16S rRNA as *Pediococcus acidilactici* strain FT28 with 100% similarity (GenBank accession nos. KU837245, KU837246 and KU837247). The *Pediococcus acidilactici* FT28 was selected as potential probiotic candidature for *in vivo* efficacy in weaned pigs. Thirty-six crossbred piglets (28d) were randomly distributed into three groups (four replicates of three each) namely, basal diet without probiotics (T0) or with *Lactobacillus acidophilus* NCDC15 (conventional dairy-specific probiotic; T1) or *Pediococcus acidilactici* FT28 (swine-specific probiotic; T2). At end of the experiment, six piglets of similar body weight were selected to conduct digestion trial for estimation of nutrient digestibility. Results of the study indicated that supplementation of both probiotics improved (P<0.001) FCR compared to control without significant effect in average daily gain and DM intake. However, the apparent digestibility of crude protein and ether extract was better (P<0.01) in pigs fed *P*. *acidilactici* FT28 compared control and *L*. *acidophilus* fed groups. The total WBC and RBC count, serum glucose, total protein, albumin and globulin concentration was higher (P<0.05) in *P*. *acidilactici* FT28 fed group with better (P<0.05) catalase and superoxide dismutase activity measured in erythrocyte. It is concluded that species-specific *Pediococcus acidilactici* FT28 isolated with potential *in vitro* probiotic properties and also hold probiotic candidature by showing the potential capabilities with higher nutrient digestibility, heamato-biochemical and antioxidant status compared to control and *Lactobacillus acidophilus* NCDC15.

## Introduction

Weaning of piglets is a natural process which occurs over several weeks or months. But due to modern practices of intensive farming, piglets are weaned early between 15–28 days of age to maximize annual sow productivity and uniform body weight at slaughter [1]. Weaning leads to abrupt change of diet from liquid to solid feed, which usually reduces the daily energy and nutrient intake thus piglets are more susceptible towards bacterial infection. However, the use of antibiotics to prevent and treat infectious diseases induce by weaning stress was banned due to emergence of antibiotic resistance and residual effect in animal products [2]. This resulted with introduction of probiotic as a live microorganism that confer a health benefit to the host, when administered in adequate amounts [3]. Probiotics not only helps in maintenance of health status in piglets have also been shown to improve growth and feed conversion ratio [4].

Many microorganisms have been used as probiotics, but lactic acid bacteria (LAB) seem to be the most potential probiotic agent in swine nutrition. Earlier studies on isolation and selection of LAB from swine intestine and feces to prepare probiotic preparation specific to swine [5, 6, 7], revealed that diet and species specificity of probiotic does exists. As the natural microflora stabilizes in the gut more easily and quickly propagates to a very stable population [8]; hence, an isolate from the host itself will be more effective probiotic as compared from other sources. For an effective probiotic agent, LAB should able to survive in gastric pH and bile salt, adhere and colonize in gut epithelial cells [9, 10, 11]. Probiotic bacteria produce organic acids, hydrogen peroxide, bacteriocin and antimicrobial compounds which may exhibit either bactericidal or bacteriostatic properties [6, 12]. To develop host specific probiotics *L*. *plantarum* and *L*. *acidophilus* (swine intestine) and *L*. *curvatus* TUCO-5E (milk) were isolated with maximum probiotic property *viz*., pH tolerance, bile tolerance, and antimicrobial activity against *E*. *coli*, *Enterobacter* spp. and Salmonella [7, 13]. Supplementation of species-specific probiotic bacteria conferred health benefits through maintenance of effective microbial balance between beneficial and harmful bacteria [6, 8, 14], enhancement of nutrient digestibility, growth and immune status in pigs [15, 16]. Feeding of LAB in weaning piglets also improved antioxidant defence mechanism (total antioxidant, superoxide dismutase, catalase, reduced glutathione and glutathione peroxidase status) thereby ameliorating oxidative stress [17, 18]. Dietary supplementation of probiotics also improved haematological profile [19] in broiler chicken, serum concentrations of total protein, albumin and globulin in weaned piglets [20], and reduced serum triglyceride and cholesterol level in pigs [21, 22]. Keeping the above background in view, the present study was conducted with an objective of isolation, phenotypic and phylogenetic characterization of LAB from piglet feces and *in-vitro* evaluation of isolated LAB for probiotic properties. To confirm species-specificity of probiotic, the effect of best performed (*in vitro*) LAB on growth performance, nutrient digestibility, health and antioxidant status in early weaned piglets by considering dairy origin *Lactobacillus acidophilus* NCDC-15 as reference [23].

## Materials and methods

This experiment was conducted in the Division of Animal Nutrition and Swine Production Farm, ICAR-Indian Veterinary Research Institute, Izatnagar, India. The animal experimental protocol was approved by Institutional Animal Ethics Committee followed by Committee for the Purpose of Control and Supervision of Experiments in Animals (CPCSEA), Ministry of Environment, Forest and Climate Change, Govt. of India.

### Isolation and culture condition of LAB

A total of 40 fecal samples were collected from five healthy piglets (28 day) of different sows for isolation of LAB. Each of the fecal samples was homogeneously mixed with sterilized phosphate buffered saline (*pH* 6.8). 1 mL of serially diluted samples were plated on Man Rogosa Sharp (MRS, Difco laboratory) agar and incubated at 37°C for 48h in BOD incubator. Transparent halo-surrounding colonies were sub-cultured in MRS broth. Subsequently, the plating and sub-culturing was done until pure colonies were obtained. Glycerol stock (30% glycerol v/v) of pure cultures were maintained at -80°C for future use.

### Biochemical and molecular identification

Each pure culture was tested for cell morphology, Gram reactions, motility and catalase activity (3% H_2_O_2_). The carbohydrate fermentation ability of the isolates was assessed by using HiCarbo^TM^ Kit part A, B and C (Cat.# KB009, Hi-Media). 50 μl of the culture (OD≥ 0.5 at 620 nm) was inoculated to each well of Hicarbo^TM^ strips by surface inoculation method. The strips were incubated at 37°C for 24h and change of colour observed in case of positive reaction (change of colour from red to yellow). Only Gram positive and catalase negative isolates were selected for further evaluation.

The molecular identification was done by amplification of 16S rRNA and rDNA gene through multiplex PCR using three primers (Table 1). The PCR mix (50 μl) contained 25 μl Dream Taq Green 2X PCR master mix (Thermo scientific), 2 μl DNA, 2 μl of each forward and reverse primers and nuclease free water. The PCR was performed by following conditions: 3 min at 95°C of initial denaturation, 35 cycles consisting of 30s at 95°C, 45s at respective annealing temperature and 1 min at 72°C and a final extension at 72°C for 7 min. The PCR product was purified using Wizard® SV Gel and PCR Clean-Up system (Cat.# A9281/2/5, Promega) and sequenced from M/s Eurofins Genomic, India Pvt. Ltd. The nucleotide sequences obtained were analyzed for homology using Basic Local Alignment Search Tool (BLAST). The reference sequences were retrieved from NCBI database and were aligned with the sequences of the isolate by Clustal W. Phylogenetic tree was constructed by 1,000 bootstrap and neighbor joining with MEGA version 5.003 [24].

**Table 1:** Primers and their sequences used for amplification of 16S rRNA and rDNA gene.

Target microbes	Sequence (5’-3’)	Annealing temp (^o^C)	Product (bp)	References
Universal bacteria	27F [AGAGTTTGATCCTGGCTCAG] 1492R [TACGGCTACCTTGTTAGGACTT]	50	1500	[25]
Lactobacillus	LpigF [TACGGGAGGCAGCAGTAG] LpigR [CATGGTGTGACGGGCGGT]	56	1200	[5]
Lactobacillus	S-17F [AGAGTTTGATCATGGCTCAG] A-17R [CACCGCTACACATGGAG]	56	700	[26]

### Resistance to different pH and bile salt

The *p*H tolerance were determined at acidic (3.0, 4.0) and basic (7.0 and 8.0) *p*H by adjusting *p*H of growth medium with 1N HCl and 1N NaOH [27]. To evaluate the resistance capacity under high bile salt (BS) condition, isolates were cultured in MRS broth with 0.075, 0.15, 0.3 and 1% (w/v) bile salts [28]. 180 μl of MRS broth of different *p*H and bile salt were inoculated with 20 μl of overnight grown cultures in 96 well microplate and incubated for 2, 4 and 6 h at 37°C. The growth curve was monitored by measuring optical density (OD) at 580 and 620 nm, respectively for pH and BS.

### Cell surface hydrophobicity

The bacterial adhesion to hydrocarbon like toluene was measured according to Palomares et al. [29]. Each culture (5 ml) was harvested in triplicate by centrifugation at 2800*xg* for 15 min, washed twice with PBS solution (pH 7.2) and re-suspended in the same buffer to have approximately 10^8^ CFU/mL (OD_600_; A). Then 2 mL of each culture suspension was placed into contact with 0.6 mL of toluene. After 10 min of incubation, bacterial suspension was thoroughly mixed with toluene by vortexing for 2 min and OD (A_0_) of aqueous phase was measured at 600nm. The hydrophobicity percentage (H) was calculated as; H (%) = [(A–A_0_)/A] x 100.

### Antimicrobial susceptibility

Antimicrobial susceptibility was determined by using antimicrobial octa-disc (Hi-Media; Combi 69, Combi VI and XIII; Cat.# OD026, OD032 and OD271). Antimicrobial octa-disc was placed after spreading the overnight grown each culture (0.1mL) onto MRS agar plates and incubated for 24-48h at 37°C. Zone of inhibition (diameter in mm) for each antibiotic was measured and expressed as susceptible, S (≥21mm); intermediate, I (16-20mm) and resistance R (≤15mm) according to Vlkova et al. [30].

### Assay of enzyme activities

The enzymatic activities of the isolates were evaluated by spot inoculation method on relevant enzymatic agar medium as described by Kim et al. [31]. The amylase activity was examined using a medium consisted with meat peptone (0.5%), yeast extract (0.7%), NaCl (0.2%), starch (2%) and agar (1.5%). For protease, isolates was inoculated over the medium consisting of skim milk (1%) and agar (1.5%). Lipase activity was detected by using a medium consisted of tryptone (0.1%), yeast extract (0.5%), NaCl (0.05%), olive oil (1%), arabic gum (1%) and agar (1.5%). The agar medium consisted of glucose (1.5%), calcium phytate (0.5%), NH_4_NO_3_ (0.5%), KCl (0.05%), MgSO_4_7H_2_O (0.05%), MnSO_4_7H_2_O (0.02%), FeSO_4_7H_2_O (0.001%), and agar (1.5%) at pH 7.0 was used for detection of phytase activity. After incubation at 37°C for 48h, the halo zone (diameter in mm) surrounding to each colony was measured with a slide caliper to assess the enzyme activity.

### Antagonistic activity against known pathogens

Antagonistic activity was assessed by using well diffusion assay [32]. The antagonistic activity was assessed against *Escherichia coli*, *Salmonella* Enteritidis, *Salmonella enteric* spp. *enteria* ser Typhimurium, *Staphylococcus intermedius*, *Staph*. *chromogenes*, *Proteus mirabillis*, *Areomonas veonii*, *Bordetella bronchiseptica* and *Klebsiella oxytoca*. The plates were incubated for 24 h at 37°C and examined for the clearing zone. The diameter (mm) of clear inhibition zone around the wells was measured with slide caliper. The size of the clearing zone was taken directly proportionate to antagonistic activity of the isolate.

### Co-culture assay

The antagonistic effect of the isolates was also used to determine through co-culture assay [33]. The 24h old grown LAB isolates and indicator pathogens were centrifuged separately at 11200*xg* for 10 min and washed twice with phosphate buffered saline (PBS). Then 3 μl of each isolates (10^8^ cfu/ml) were co-incubated with 3 μl any of the seven pathogens including *E*. *coli*, *Salmonella enteric* spp. *enteria* ser Typhimurium, *Staph*. *intermedius*, *Staph*. *chromogenes*, *P*. *mirabillis*, *A*. *veonii* and *K*. *oxytoca* (10^5^ cfu/ml), respectively, in 3 ml nutrient broth and incubated at 37°C for 24h. Serially diluted (10^−6^) 1ml culture was plated on nutrient agar plates and incubated for 24h at 37°C. The colonies of each pathogen were counted and the counts were compared with respective monoculture of pathogen as control. The inhibition percentage was calculated according to the following equation [34].

$$\text{Inhibition }\left(\text{\%}\right)\text{ =}\frac{{\text{CFU ml}}^{\text{-1}}{\text{in control – CFU ml}}^{\text{-1}}\text{in co-incubation culture}}{\left({\text{CFU ml}}^{\text{-1}}\text{in control}\right)}\text{X 100}$$

### *In-vitro* adhesion to intestinal epithelium

The adhesion to intestinal epithelium was assessed according to Kos et al. [35]. Pig and chicken intestinal samples were collected and suspended in PBS solution at 4°C for 30 min; then washed thrice with PBS. The suspension (10^9^ cells/ml PBS) of the isolate was incubated with intestinal sample (1 cm^2^) for 30 min at 37°C. The intestinal samples were fixed in 10% formalin, dehydrated and embedded in paraffin. Serial sections (5 μm) were cut, and stained with haemotoxylin and eosin. The slides were examined and photographed at 100× and 400× magnifications.

### *In-vivo* evaluation of selected LAB in weaned piglets

A total of 36 (18 male: 18 female) early weaned (28 day old) crossbred (Local x Landrace) piglets were used for the *in-vivo* experiment. The piglets were distributed into three dietary groups (4 replicates of 3 each) in complete randomized design. Dietary treatments included; T0 (control-basal diet), T1 (basal diet + *Lactobacillus acidophilus* NCDC15; conventional dairy-specific probiotic) and T2 (basal diet + *Pediococcus acidilactici* FT28; swine-specific probiotic). The piglets were maintained under uniform conditions by housing them in well-ventilated pans with feeder and water tough facilities. All piglets were dewormed with Albendazole oral suspension at the beginning of the experiment and protected against swine fever by using attenuated swine fever vaccines. The feeding experiment was conducted for 90 days period.

### Experimental diet and feeding regime

The basal diet was formulated as per NRC [36] recommendation (Table 2). The probiotics were supplied through fermenting the ground maize with either of the overnight culture, which was prepared as per the method described by Agarwal et al. [37] and fed @ 200g/day/head. Probiotic product (containing 2.0 x 10^9^ cfu/g) was mixed into the basal diet in the morning (0900 h) by subtracting the equal amount of maize. Body weight of each animal was recorded at weekly interval and the feeding schedule was adjusted accordingly. Clean fresh drinking water and feed were given *ad lib*. throughout the experiment.

**Table 2:** Ingredient and chemical composition (% DM) of the experimental basal diets.

Attributes	Body weights (kg)			
	5–10	10–20	20–50
***Ingredient composition***			
Crushed maize	46	54	62
Deoiled soybean meal	30	22	15
Wheat bran	16	16	15
Fish meal	06	06	06
Mineral mixture*	1.5	1.5	1.5
Common salt	0.5	0.5	0.5
***Chemical composition***			
DM	90.20	90.25	90.40
OM	91.27	91.66	92.37
CP	23.22	20.99	18.59
EE	2.39	3.09	3.80
CF	4.31	4.55	4.72
Total ash	8.73	8.34	7.63
NFE	61.35	63.03	65.26
Calcium	0.82	0.71	0.62
Phosphorous	0.63	0.61	0.55
GE (Mcal/kg)	4.16	4.17	4.17

*Each 250 g contain: vitamin A 5,00,000IU; vitamin D3 1,00,000IU; vitamin B2 0.2g; vitamin E 75mg; vitamin K 0.1g; vitamin B12 600mcg; calcium pantothenate 25mg; niacin 1g; choline chloride 15g; calcium 70g; manganese 2.75g; iodine 0.1g; iron 0.75g; zinc 1.5g; copper 0.2g; cobalt 0.045g; phosphorus 20g.

### Growth performance and nutrient digestibility

The growth performance was monitored throughout the experimental period of 90 days. Daily feed offered and refused was weighed and recorded to monitor daily dry matter intake (DMI). Piglets were weighed individually at weekly intervals to calculate growth parameters *viz*. average daily gain (ADG) and feed conversion ratio (FCR). At end of the feeding experiment, a digestion trial was conducted for 7 days (3 days adaptation + 4 days collection) to know digestibility of the nutrients. Six animals (3 male and 3 female) from each group of comparable body weight were transferred to individual floor pen with run. The feed offered and residue left were collected daily and pooled over for four days and sub-samples were taken for further analysis. The faeces from each animal were collected manually just after defecation, and pooled for 24h and weighed daily at 9.00 AM. Ten percent of the faecal samples were collected daily and stored at -20°C and pooled samples were taken for estimation of nitrogen. Another ten percent faecal samples from each animal were collected daily and dried in hot air oven at 60±5°C for 24–48 h for estimation of DM. The dried samples were ground in a hammer mill to a fineness of 1mm size and analysed for proximate principles [38].

### Blood haemato-biochemical profile

Blood samples were collected from cranial vena cava in the morning (before watering and feeding) into anti-coagulated vaccutainer tubes from all the pigs at 45 and 90 day of feeding trial. The haematological profile *viz*. haemoglobin (Hb), red blood cell (RBC) and white blood cell (WBC) of the whole blood was analyzed with Haematology Analyser by Clindiag system B.V.B.A (Cat. # HA-22/20/Vet) according to manufacturer condition. Serum was separated from whole blood by centrifugation at 3000 *xg* for 30 min. The metabolites like glucose, total protein, albumin and triglycerides was determined colorimetrically using commercial diagnostic kits (Span Diagnostics Pvt. Ltd., India) by spectrophotometer model UV-2601, Labomed, USA.

### Erythrocytic antioxidant profile

Blood samples were collected into vaccutainer tubes with acid citrate dextrose (300 μl per 2ml blood) from all the pigs at 45 and 90 day of feeding by puncturing cranial vena cava before watering and feeding in the morning. Superoxide dismutase (SOD) in the haemolysate was estimated by using nitro blue tetrazolium as a substrate after suitable dilution as per the method of Marklund and Marklund [39] with certain modifications [40]. Catalase was analyzed by the spectrophotometric method using H_2_O_2_ as a substrate [41]. Reduced glutathione (GSH) activity in packed RBC was measured by 5, 5-dithiobis-(2-nitro-benzoic acid; DTNB) method as per the procedure of Prins and Loos [42]. Where, the haemolysate and RBC suspension were prepared from whole blood as per the standard protocol [43].

### Statistical analysis

The data generated for *in-vitro* probiotic profile, growth performance, blood haemato-biochemical and antioxidant profile were analyzed by using following general linear model;

$$\begin{array}{l} y_{ij}=\mu \;\;+\;\;T_{i}+\;\;e_{ij}\\ Where\\ y_{ij}=\;Observed\;value\;of\;the\;response\;variable\;for\;i^{th}\;group\;and\;j^{th}\;observation\;\\ µ=\;General\;mean\;effect\\ T_{i}=Effect\;of\;i^{th}\;treatment\;\\ e_{ij}\;=\;error\;term\;which\;is\;normally\;distributed\;with\;mean\;0\;and\;variance\;\;\sigma^{2}\;\;\;\; \end{array}$$

Duncan’s Multiple Range Test was used for comparison between different pairs of treatments [44]. The effects were considered to be significant at 5% level of significance (P<0.05*)*. Analysis was done by using SPSS (version 17.0 for Windows; SPSS, Chicago, III., U.S.A.) software.

## Results

### Isolation and characterization of LAB

The isolates were cocco-bacilli, Gram-positive, catalase-negative and occurred either in single or cluster of three to five cells with creamy white colonies in MRS agar, which confirmed that all of them belong to LAB group. These isolates were further screened for their *in-vitro* probiotic properties. All the isolates were utilized twenty-seven carbohydrates *viz*. dextrose, lactose, arabinose, galactose, fructose, glycerol, maltose, mannose, cellobiose, dulcitol, sorbitol, sorbose, sucrose, trehalose, xylose, xylitol, adonitol, arabitol, d-methyl-d monnoside, erythritol, raffinose, rhamnose, inositol, inulin, manitol and melibiose except ONPG, citrate and malonate (S1 Table). No significant differences were evident among the isolates.

### Resistance to pH and bile salt

**Fig 1:**
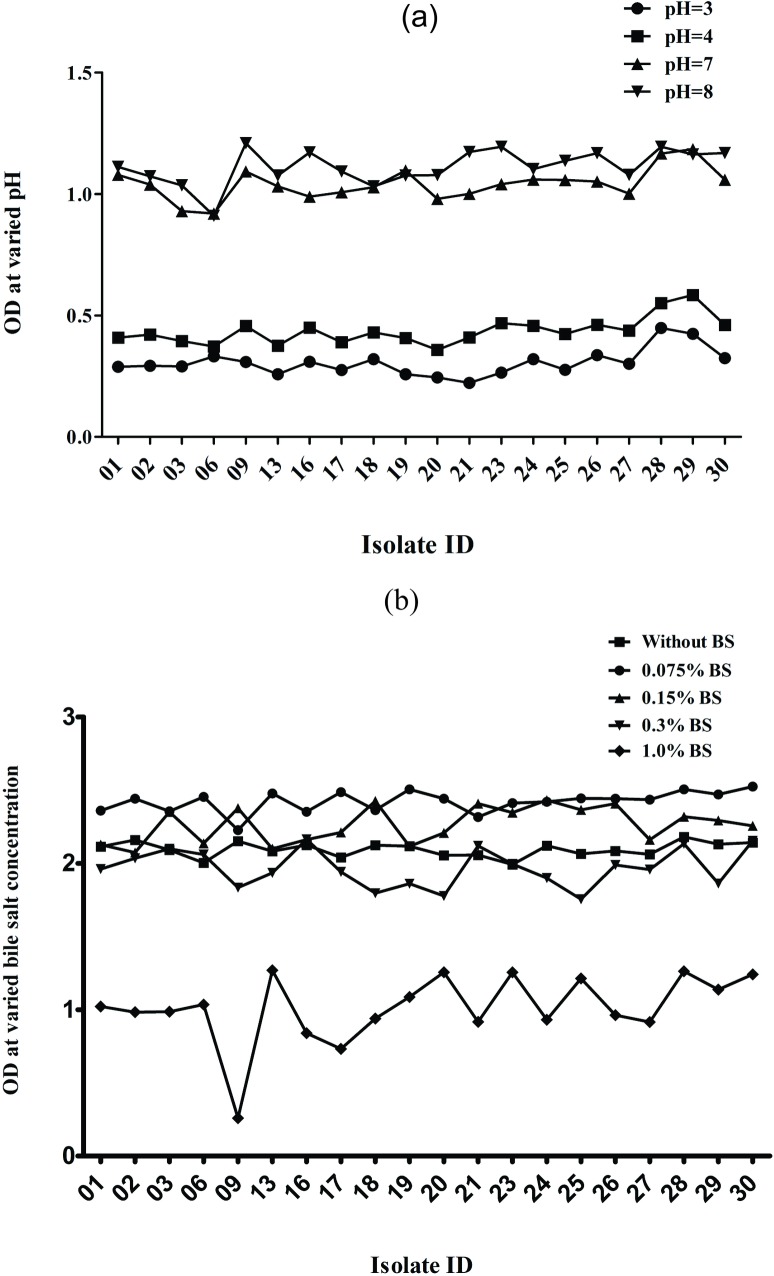
Growth of the LAB isolates (n = 20) at varied pH and bile salt, respectively after 6h incubation at 37°C.

Out of thirty isolates, twenty were enabled to survive at different *p*H (3.0, 4.0, 7.0 and 8.0) and bile salt (0.075, 0.15, 0.5 and 1.0%) at 37°C for 2, 4 and 6h (Fig 1). Whereas, rest of the isolates either showed no growth or very weak growth therefore were discarded. Among the selected isolates, Lacp28 and Lacp29 showed highest (P<0.001) optical density in *p*H 3.0, 4.0 and 7.0 but in *p*H 8.0, Lacp09 exhibited maximum OD (Fig 1). Inclusion of BS in MRS broth upto 0.15% stimulated growth of the LAB as compared to control (without BS) except few which were at par with the control (Fig 1). Presence of BS upto 1.0% in growth medium was highly detrimental whereas, 0.3% inhibited growth of the majority of LAB isolates.

### Cell surface hydrophobicity

**Fig 2:**
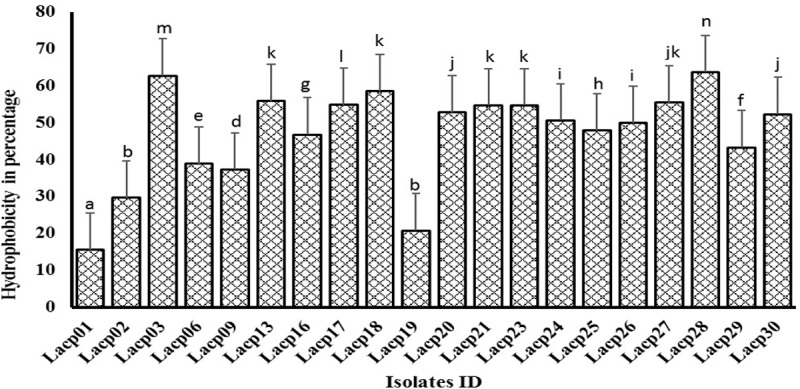
Cell surface hydrophobicity assay (%) of LAB isolates.

The isolates showed a wide range of hydrophobicity ranging from 15.50 to 63.70% (Fig 2). The Lacp28 isolate exhibited highest (P<0.05) hydrophobic activity against toluene. Out of twenty isolates, six (Lacp 01, 02, 06, 09, 19, 29) were showed less than 45% cell surface hydrophobicity, for this reason those were discarded and the further characterization was proceeded with remaining isolates.

### Enzymatic activity

The size of the halo zones was directly proportionate to the enzyme activity. All the isolates were enabled to synthesized enzymes *viz*. amylase, protease, lipase and phytase. Whereas, diameter of the halo zone for amylase and lipase was not differed (P>0.05) among the isolates. Maximum (P<0.05) halo zone was attributed to Lacp23, Lacp25, Lacp26 and Lacp28 for protease, and Lacp28 for phytase (Table 3).

**Table 3:** Enzymatic activities of LAB isolates in different agar medium (at 37°C for 48h).

Isolates	Diameter of halo zone^†^ (mm)			
	Amylase	Protease	Lipase	Phytase
Lacp03	4.75	4.75^b^	10.75	16.50^a^
Lacp13	5.00	0.00^a^	10.00	17.75^ab^
Lacp16	5.50	6.75^c^	10.25	17.25^ab^
Lacp17	5.75	0.00^a^	10.00	20.00^cd^
Lacp18	5.75	8.00^de^	11.50	16.50^a^
Lacp20	4.75	8.75^e^	10.50	15.75^a^
Lacp21	5.50	8.00^de^	10.25	19.00^bc^
Lacp23	5.00	10.50^f^	10.75	16.00^a^
Lacp24	5.25	7.25^cd^	10.75	17.50^ab^
Lacp25	6.00	10.25^f^	10.50	16.25^a^
Lacp26	5.50	10.00^f^	10.75	16.25^a^
Lacp27	6.00	8.50^e^	11.25	17.00^ab^
Lacp28	6.25	9.75^f^	10.25	21.00^d^
Lacp30	6.50	8.50^e^	10.25	20.50^cd^
SEM	0.13	0.45	0.09	0.27
P value	0.293	<0.001	0.069	<0.001

† Mean of three observations each

^abcdef^Means bearing different superscripts in a column differ significantly (P<0.05)

### Antimicrobial susceptibility

All the selected isolates were resistant (diameter ≤15mm) to ciprofloxacin, ofloxacin, gatifloxacin, vancomycin and co-trimoxazole and sensitive (diameter ≥21mm) to penicillin, azithromycin, clindamycin, erythromycin, cephalothin and chloramphenicol (Table 4).

**Table 4:** Resistance of the selected LAB isolates to various antibiotics (Hi-Media).

Antibiotics^†^	Disc content	Isolates No													
		*Lacp03*	*Lacp13*	*Lacp16*	*Lacp17*	*Lacp18*	*Lacp20*	*Lacp21*	*Lacp23*	*Lacp24*	*Lacp25*	*Lacp26*	*Lacp27*	*Lacp28*	*Lacp30*
CIP	5 μg	R	R	R	R	R	R	R	R	R	R	R	R	R	R
OF	5 μg	R	R	R	R	R	R	R	R	R	R	R	R	R	R
GAT	5 μg	R	R	R	R	R	R	R	R	R	R	R	R	R	R
P	2 unit	S	S	S	S	S	S	S	S	S	S	S	S	S	S
AZM	15 μg	I	S	S	S	S	S	S	S	S	S	S	I	S	I
L	10 μg	S	S	S	S	S	S	S	S	S	S	S	S	S	S
VA	30 μg	R	R	R	R	R	R	R	R	R	R	R	R	R	R
CD	2 μg	S	S	S	S	S	S	S	S	S	S	S	S	S	S
E	10 μg	S	S	S	S	S	S	S	S	S	S	S	S	S	S
GEN	10 μg	R	I	I	R	I	I	I	I	I	I	I	I	I	I
TE	10 μg	S	I	I	I	I	I	S	I	I	I	I	S	I	S
DO	30 μg	I	I	I	I	I	I	I	I	I	I	I	I	I	I
COX	5 μg	I	R	R	I	R	R	I	R	I	I	I	I	I	I
AMP	10 μg	I	I	I	I	S	I	I	I	I	I	I	I	S	I
OX	1 μg	I	I	I	S	I	I	I	I	I	I	I	I	I	S
COT	25 μg	R	R	R	R	R	R	R	R	R	R	R	R	R	R
CXM	30 μg	S	R	I	I	I	I	I	R	I	I	I	I	R	I
CEP	30 μg	S	I	I	S	S	S	S	S	S	S	S	S	S	S
C	30 μg	S	S	S	S	S	S	S	S	S	S	S	S	S	S

^**†**^CIP, ciprofloxacin; OF, ofloxacin; GAT, gatifloxacin; P, penicillin-G; AZM, azithromycin; L, lincomycine; VA, vancomycin; CD, clindamycin; E, erythomycine; GEN, gentamycin; TE, tetracycline; DO, doxycycline hydrochloride; COX, cloxacillin; AMP, ampicillin; OX, oxacillin; COT, co-trimoxazole; CH, cefradine; CXM, cefuroxime; CEP, cephalothin; C, chloramphenicol

### Antagonistic activity against known pathogens

The isolates showed antagonistic activity against all the nine pathogens tested except, isolate Lacp03 and Lacp30, and Lacp27 which did not show any inhibition zone against *B*. *brochiseptica* and *A*. *veonii*, respectively (Table 5). The Lacp28 isolate displayed highest (P<0.001) antagonistic activity against *E*. *coli*, *S*. Enteritidis, *Staph*. *intermedius*, *Staph*. *chromogenes*, *P*. *mirabillis* and *A*. *veonii*, whereas, Lacp30 showed maximum (P<0.001) inhibitory zone against *E*. *coli*, *P*. *mirabillis* and *K*. *oxytoca*. Moreover, *E*. *coli*, *S*. Typhimurium and *B*. *bronchiseptica* growth were significantly (P<0.001) inhibited by Lacp16, Lacp21 and Lacp24, and Lacp25, respectively.

**Table 5:** Antagonistic activity (diameter of inhibition zone; mm) of selected LAB isolates against pathogenic bacteria (at 37°C for 24h).

**Isolates No**	Diameter of inhibition zone^†^ (mm)								
	*E*. *coli*	*Salmonella* Typhimarium	*S*. Enteritidis	*Staph*. *intermedius*	*Staph*. *chromogenes*	*Proteus mirabillis*	*Bordetella brochioseptica*	*Areomonas veonii*	*Klebsialla oxytoca*
Lacp03	10.33^a^	12.33^cd^	13.67^defg^	11.00^ab^	10.33^a^	9.67^a^	0.00^a^	9.00^b^	14.33^e^
Lacp13	13.33^cd^	11.67^abc^	12.00^bc^	15.00^cde^	13.67^cdef^	12.33^bc^	12.33^cd^	13.67^fg^	8.00^a^
Lacp16	13.67^d^	10.67^a^	13.00^bcdef^	16.33^def^	12.67^bc^	10.33^a^	14.67^g^	12.33^def^	12.00^bcd^
Lacp17	12.67^cd^	12.00^abc^	11.67^b^	16.67^def^	14.33^defgh^	12.67^cd^	13.33^de^	13.33^efg^	10.67^b^
Lacp18	12.00^abc^	12.00^abc^	12.33^bcd^	13.00^bc^	13.33^bcde^	10.00^a^	13.67^e^	11.00^cd^	10.67^b^
Lacp20	12.00^abc^	12.00^abc^	13.33^cdefg^	16.00^cde^	13.00^bcd^	13.00^cd^	10.67^b^	11.67^de^	10.33^b^
Lacp21	12.00^abc^	13.00^d^	14.00^efg^	9.33^a^	15.33^gh^	13.67^cde^	12.33^cd^	10.67^cd^	10.33^b^
Lacp23	11.67^ab^	12.67^d^	13.67^defg^	11.33^ab^	12.00^b^	15.00^e^	12.00^c^	12.33^def^	11.00^bc^
Lacp24	12.67^cd^	13.00^d^	14.67^gh^	14.00^bcd^	13.33^bcde^	13.00^cd^	14.00^ef^	12.00^def^	12.67^cde^
Lacp25	12.00^abc^	12.67^d^	14.33^fgh^	17.67^ef^	14.00^cdefg^	11.00^ab^	14.67^g^	10.00^bc^	11.00^bc^
Lacp26	13.67^d^	11.67^abc^	12.67^bcde^	14.00^bcd^	15.00^fgh^	13.33^cd^	13.00^cde^	12.00^def^	12.00^cd^
Lacp27	12.67^cd^	11.00^ab^	13.67^defg^	11.00^ab^	14.67^efgh^	10.33^a^	10.00^b^	0.00^a^	10.67^b^
Lacp28	13.67^d^	12.6^d^	15.67^h^	22.67^g^	15.67^h^	14.00^de^	13.00^cde^	14.00^g^	13.67^de^
Lacp30	11.67^ab^	11.00^ab^	10.33^a^	19.33^f^	15.33^gh^	14.00^de^	0.00^a^	10.67^cd^	14.00^e^
SEM	0.17	0.13	0.22	0.58	0.23	0.27	0.73	0.52	0.27
P value	<0.001	<0.001	<0.001	<0.001	<0.001	<0.001	<0.001	<0.001	<0.001

^†^Mean of three observations each

^abcdefgh^Means bearing different superscripts in a column differ significantly (P<0.01)

### Co-culture assay

All fourteen isolates showed more than 60 percent growth inhibition (co-culture activity) seven different known pathogens (Table 6). Best (P<0.001) co-culture activity (>80% growth inhibition) was observed with Lacp28 against *E*. *coli*, *S*. Typhimurium, *Staph*. *intermedius*, *Staph*. *chromogenes*, *P*. *mirabillis* and *A*. *veonii;* whereas, *K*. *oxytoca* growth was maximum (P<0.001) inhibited by Lacp03 and Lacp30 compared to other isolates.

**Table 6:** Co-culture assay (inhibition percentage) of LAB isolates against various pathogens (at 37°C for 24h).

Isolate Nos.	Growth inhibition of pathogenic bacteria^†^ (%)						
	*E*. *coli*	*Salmonella* Typhimarium	*Staph*. *intermedius*	*Staph*. *chromogenes*	*Proteus mirabillis*	*Areomonas veonii*	*Klebsialla oxytoca*
Lacp03	52.90^a^	75.31^e^	66.13^b^	58.98^a^	57.20^a^	72.11^b^	76.19^j^
Lacp13	73.33^e^	69.63^bcd^	75.47^e^	75.64^d^	67.69^cd^	87.30^h^	48.94^b^
Lacp16	77.63^f^	81.23^g^	76.80^e^	62.82^b^	62.55^b^	81.18^f^	61.64^g^
Lacp17	66.24^d^	70.86^d^	77.07^ef^	79.49^f^	67.28^c^	83.22^g^	43.39^a^
Lacp18	62.58^c^	70.62^d^	69.33^c^	68.72^c^	62.14^b^	75.28^c^	53.70^de^
Lacp20	62.15^c^	70.86^d^	75.20^de^	64.36^b^	69.34^de^	77.78^d^	51.59^c^
Lacp21	62.15^c^	82.22^g^	64.00^a^	83.08^g^	70.99^ef^	74.60^c^	52.38^cd^
Lacp23	56.56^b^	76.54^ef^	66.40^b^	64.36^b^	75.93^g^	81.86^fg^	58.73^f^
Lacp24	67.74^d^	64.44^a^	73.33^d^	74.36^d^	72.016^f^	78.68^de^	65.61^h^
Lacp25	63.01^c^	77.28^f^	80.27^h^	77.69^e^	67.28^c^	72.34^b^	60.32^fg^
Lacp26	79.35^f^	70.37^cd^	75.20^de^	82.82^g^	71.19^ef^	79.37^e^	64.55^h^
Lacp27	65.81^d^	68.64^bc^	66.13^b^	80.51^f^	63.17^b^	68.71^a^	54.50^e^
Lacp28	83.01^g^	80.49^g^	86.93^i^	87.44^i^	74.07^g^	90.48^i^	73.016^i^
Lacp30	56.56^b^	68.15^b^	78.93^gh^	84.36^g^	74.49^g^	75.06^c^	74.87^j^
SEM	1.37	0.84	0.99	8.99	0.83	0.92	1.51
P value	<0.001	<0.001	<0.001	<0.001	<0.001	<0.001	<0.001

^†^Mean of three observations each

^abcdefghij^Means bearing different superscripts in a column differ significantly (P<0.05)

### Molecular identification of selected strain

**Fig 3:**
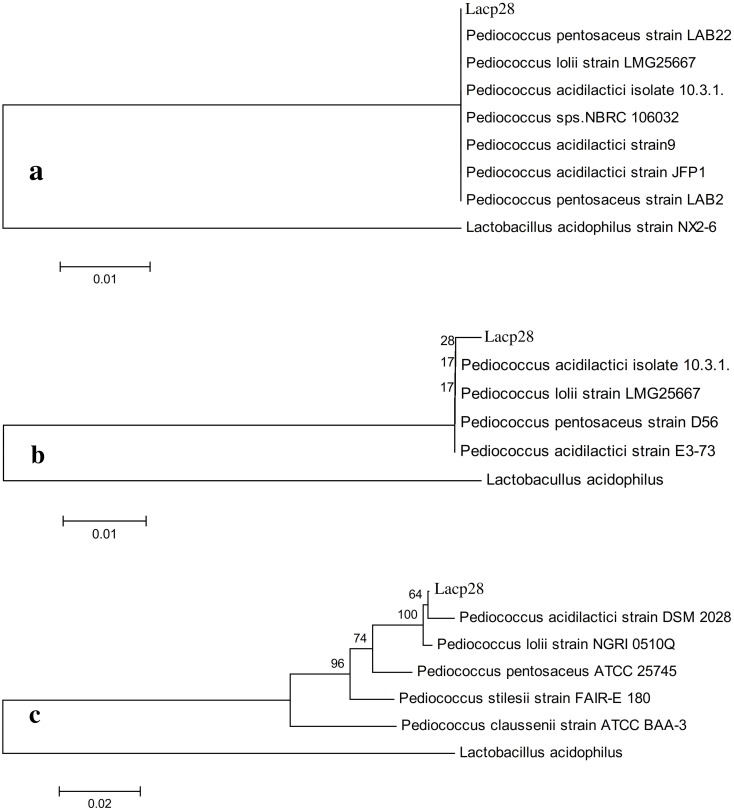
Phylogenetic tree prepared by Clustal V method for molecular identification of Lacp28 with different primers a) 27F/1492R; b) LpigF/LpigR and c) S-17F/ A-17R.

Based on results, the Lacp28 isolate was selected for molecular identification. Since, Lacp28 showed the best probiotic properties in terms of survivability in acidic *p*H (P<0.001), tolerance to bile salt upto 0.3%, cell surface hydrophobicity (*P*<0.05), and antimicrobial activity (*P*<0.001). The PCR amplicons of 1500, 1200 and 700 bp were obtained with 27F/1492R, LpigF/LpigR and S-17-F/A-17-R primers, respectively (S1 Fig). The phylogenetic analysis of 16S rRNA gene sequence (GenBank accession No KU837245) with 27F/1492R primer showed a single cluster with various *Pediococcus sp*. having 100% similarity (Fig 3) without any distance indicating that Lacp28 isolate belonged to genus *Pediococcus*. Amplification of 16S rDNA with other two primers *i*.*e*. LpigF/LpigR (GenBank accession No. KU837246) and S-17-F/A-17-R (GenBank accession No. KU837247) confirmed that Lacp28 was from the genus *Pediococcus* and since in both the phylogenetic tree (Fig 3) showed closest to *Pediococcus acidilactici* strain (99% similarity), therefore Lacp28 was identified as *Pediococcus acidilactici* and was named as *Pediococcus acidilactici* strain FT28.

### *In vitro* adhesion to intestinal epithelium

**Fig 4:**
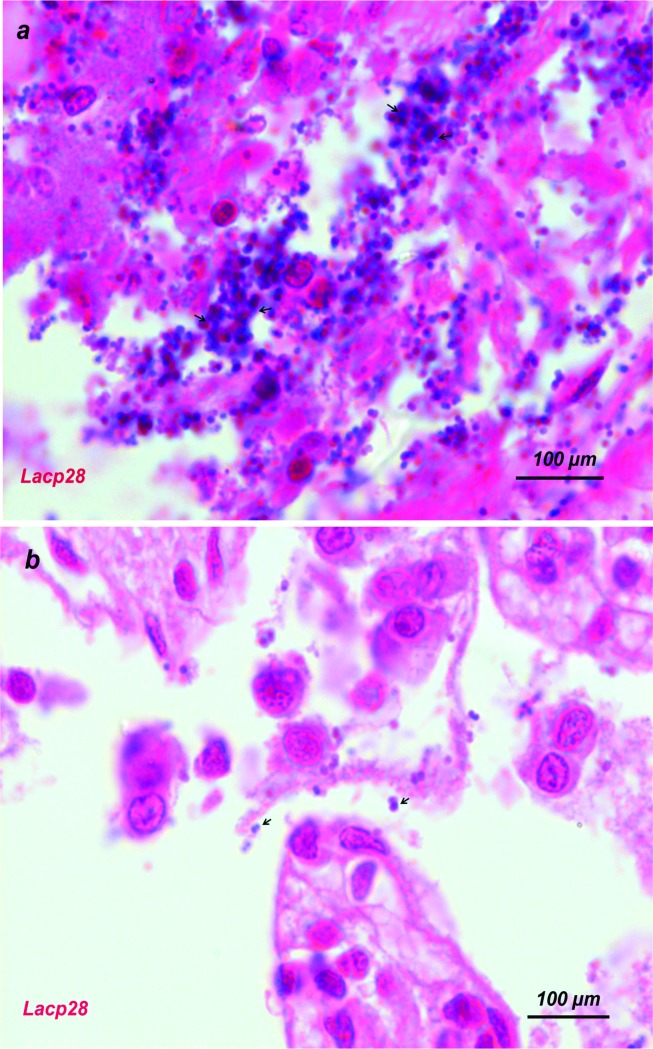
*In vitro* adhesion of the isolates (Lacp28) to epithelium cells of pig's ileum (a) and chicken intestine (b) after treatment with cell suspension (1x10^9^ cfu/ml). Magnification, X400.

The *Pediococcus acidilactici* FT28 (Lcap28) showed heavy *in vitro* adhesion towards pig intestinal epithelial cells compared to control (Fig 4) in 400X magnification. However, no adhesion was visible to chicken intestinal epithelial cells (Fig 4).

### Growth performance and nutrient digestibility

The final body weight, average daily gain (g/d), DM intake (g/d) was not differed (P>0.05) among the treatment groups (Table 7). Whereas, the feed conversion ratio was improved (P<0.05) in both probiotics supplemented (T1 and T2) groups compared to control (T0). The total tract apparent digestibility of crude protein was significantly higher (P<0.05) in T2 animals which were fed *P*. *acidilactici* FT28 as compared to T0 and T1 (Table 7). The crude fibre digestibility was higher (P<0.05) higher in T2 group in comparison to T0, whereas, T1 was comparable to T2 and T0 both, whereas, there was no effect on dry matter, organic matter and ether extract digestibility.

**Table 7:** Effect of probiotics on growth performance and nutrient digestibility in early weaned crossbred pigs.

Weeks	Dietary treatments^†^			SEM	P value
	T0	T1	T2		
***Growth performance***					
IBW (kg)	7.43	7.05	8.06	0.23	0.215
FBW (kg)	44.08	44.83	47.35	0.92	0.362
ADG (g/d)	402.8	415.2	431.7	8.96	0.429
DMI (g/d)	1023	936.7	928.8	21.0	0.124
FCR	2.55^b^	2.26^a^	2.16^a^	0.06	0.010
***Apparent digestibility (%)***					
Dry matter	82.85	80.83	81.55	1.13	0.686
Organic matter	81.85	81.46	83.08	0.61	0.556
Crude protein	74.85^a^	75.96^a^	81.86^b^	1.08	0.008
Crude fibre	30.24^a^	35.96^ab^	50.24^b^	3.29	0.042
Ether extract	59.93	62.02	67.28	3.30	0.308

^**†**^ No probiotics (T0), *L*. *acidophilus* (T1) and *P*. *acidilactici* FT28 (T2)

^ab^ Means with different superscript within a row differ significantly. IBW; initial body weight, FBW; final body weight, ADG; Average daily gain, DMI; dry matter intake, FCR; feed conversion ratio

### Blood haemato-biochemical profile

**Fig 5:**
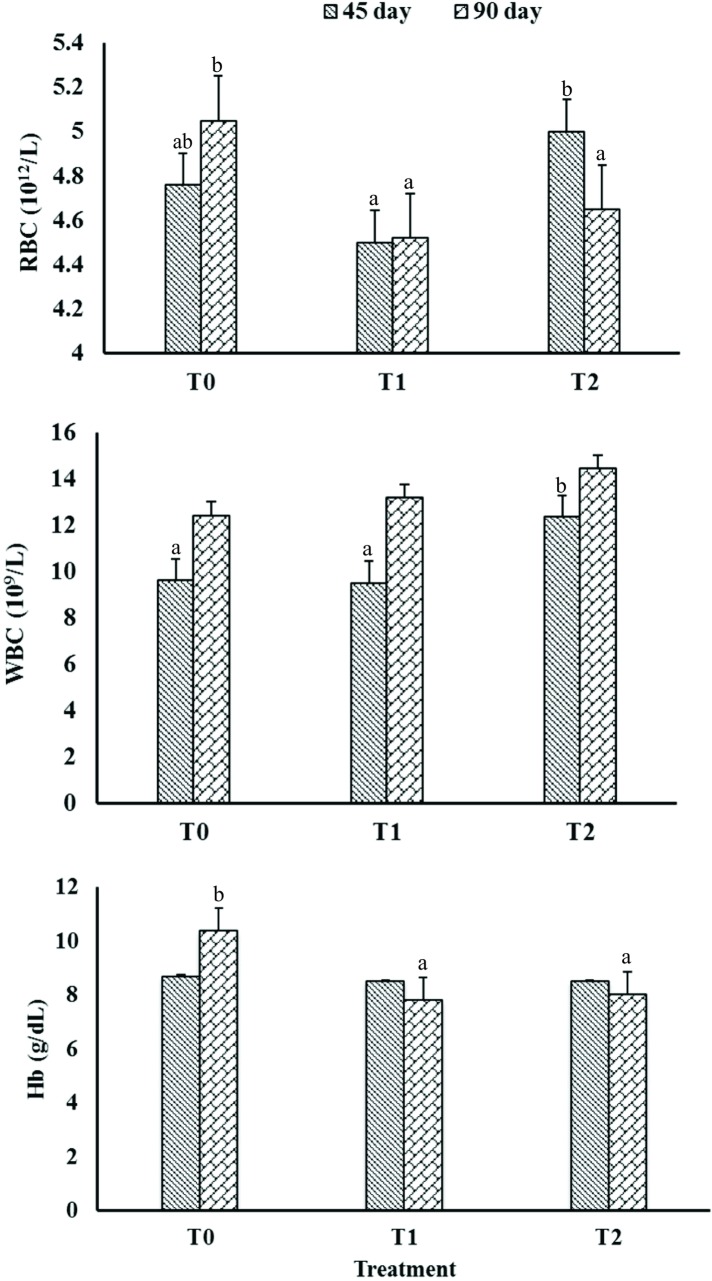
Effect of probiotics on red blood cells (RBC; 10^12^/L), white blood cells (WBC; 10^9^/L) and haemoglobin (Hb; g/dL) percentage in crossbred weaning pigs.

In day 45, the RBC count was higher (P<0.05) in T2 group compared to T1, where T0 showed intermediate value (Fig 5). However, T1 and T2 groups showed lower (P<0.05) RBC count compared to T0 in day 90. The WBC count was enhanced (P<0.05) in *P*. *acidilactici* FT28 fed animals (T2 group) in day 45. Whereas, WBC and Hb concentration were not differed among the treatment groups in day 90 and 45, respectively. The later was significantly higher (P<0.001) in control compared to both supplemented groups in day 90.

Serum level of glucose was highest (P<0.001) in supplemented group compared to control, where *P*. *acidilactici* FT fed animals had better serum glucose than *L*. *acidophilus* fed group. Total protein concentration was highest (P<0.001) in T2 group compared to T0 and then T1 groups. Serum albumin and globulin concentration were enhanced (P<0.001) in T2 groups compared to other groups. Where, the concentration of albumin was statistically similar in T0 and T2. Due to effect of period and interaction treatment *x* period also showed significant (P<0.05) results for serum glucose and albumin. However, the levels of triglyceride did not showed any significant (P<0.05) effect between the groups.

### Erythrocytic antioxidant profile

**S**upplementation of basal diet with probiotics improved (P<0.05) GSH activity in packed RBC and catalase concentration in erythrocytes compared to control (Table 8). Whereas, the interaction of treatment *x* period showed significant effect on GSH activity. The concentration of SOD in erythrocytes was increased (P<0.05) in better (P<0.05) in *P*. *acidilactici* FT28 fed (T2) animals than *L*. *acidophilus* (T1) supplemented pigs, where T1 group showed intermediate values.

**Table 8:** Effect of feeding different probiotic source in blood biochemical and antioxidant profile on early weaned crossbred pigs.

Treatment^†^	Period		Mean	SEM	Significance^‡^		
	Day 45	Day 90			T	P	T*P
***Blood biochemical profile***							
*Glucose (mg/dL)*							
T0	71.75	65.91	68.85^a^	3.29	<0.001	0.010	0.015
T1	68.06	89.03	78.54^b^	3.29			
T2	91.63	107.8	100.3^c^	3.41			
*Total protein (g/dL)*							
T0	5.65	6.08	5.87^b^	0.19	<0.001	0.017	0.106
T1	5.07	5.13	5.10^a^	0.19			
T2	7.65	8.79	8.26^c^	0.19			
*Albumin (g/dL)*							
T0	3.67	3.42	3.55^b^	0.10	<0.001	0.017	0.006
T1	2.57	2.97	2.77^a^	0.10			
T2	3.29	3.99	3.66^b^	0.10			
*Globulin (g/dL)*							
T0	1.98	2.66	2.32^a^	0.20	<0.001	0.378	0.100
T1	2.61	2.10	2.35^a^	0.20			
T2	4.36	4.82	4.60^b^	0.21			
*Triglyceride (mg/dL)*							
T0	46.83	38.75	42.79	3.08	0.287	0.064	0.075
T1	46.61	29.96	38.28	3.08			
T2	33.69	37.93	35.95	3.19			
***Erythrocytic antioxidant profile***							
*Reduced glutathione (mg/100 ml packed RBC)*							
T0	1.26	1.20	1.23^a^	0.05	0.001	<0.001	<0.001
T1	1.19	1.73	1.46^b^	0.05			
T2	1.34	1.69	1.53^b^	0.05			
*Catalase (Unit/mg Hb)*							
T0	60.99	49.02	55.01^a^	4.00	0.030	0.227	0.639
T1	68.41	65.74	67.08^b^	4.00			
T2	74.15	68.62	69.80^b^	4.14			
*Superoxide dismutase (U/mg Hb)*							
T0	71.24	131.6	101.4^a^	5.59	0.018	<0.001	0.421
T1	74.42	155.5	114.9^ab^	5.59			
T2	91.67	159.1	127.6^b^	5.79			

^**†**^ No probiotics (T0), *L*. *acidophilus* (T1) and *P*. *acidilactici* FT28 (T2)

^abc^Means with different superscript within a column differ significantly

^‡^Significant effects of dietary treatment (T), period (P) or their interaction (T*P)

## Discussion

Along with other probiotic properties, the host origin microbes are preferred as they quite familiar with GIT environment and can proliferate and express its biological activity in a better way as compared to the microbes which are from any other source. Therefore, there is need to develop species-specific probiotic for better health and performance of livestock.

In the present study, thirty Gram-positive, catalase-negative and non-sporulated, cocco-bacilli occurred in single or cluster of three to five cells with creamy white colonies in MRS agar, which () primarily confirmed that all are under the Lactobacteriaceae family. The present findings are in agreement with previous report for *Lactobacilus ruminis* isolated from GIT of the healthy pig [45]. These isolates were also enabled to utilize twenty-seven different carbohydrates except ONPG, citrate and malonate. Though it is difficult to differentiate among the isolates, the test can be used for probable bacteriological classification only [46]. Du Toit et al. [47] also reported similar carbohydrate fermentation profiles for *L*. *reuteri* and *L*. *buchneri* (isolates of pig feces), with no clear distinction between the two phenotypes and genotypes. Similarly, LAB isolates (n = 24) showed positive reaction by fermenting lactose, fructose, galactose, trehalose, mellobiose, manose, xylitol and sorbose, and none of the isolates could utilized ONPG [48].

Acid and bile salts (around 0.3%) tolerance is one of the important criteria for the selection of probiotic strain as these are the few major factors dictating the probability of survival of an exogenous culture in the GIT [49]. Therefore, ten isolates showing very poor tolerance to low pH and to presence of bile salts were discontinued. During the passage through stomach, the probiotic microbes have to survive at low *p*H as of 3.0 before reaching in lower tract and must remain viable for 4h or more [50], therefore Lacp28 and Lacp29 can be considered to be best as they had higher growth at acidic *p*H (3.0 and 4.0). Du Toit et al. [27] reported that *L*. *reuteri* BFE1058 and *L*. *johnsonii* BFE1061 isolated from pig feces were able to grow at low *p*H of 3.0 and 4.0 for 6h at 37°C. The cultures of *L*. *lactis* and *Enterococcus faecium* exhibited better tolerance to low *p*H than *L*. *casei* and *P*. *acidilactici*, therefore were used for feeding to weaned piglets as probiotics [51]. For an effective probiotic culture, LAB must be viabled at around 0.3% bile salts [28]. In the present study also, the isolate Lacp03, Lacp06, Lacp21, Lacp28 and Lacp30 were enabled to tolerate BS upto 0.3%. An isolate, *L*. *plantarum* ZlP001 from GIT of weaned piglet showed 85.3, 61.4 and 9.4% tolerance when exposed to 0.1, 0.3 and 0.5% bile salts in the growth media [15]. Lacp29 showed better resistance at acidic pH but unable to survive at 0.3% BS.

The hydrophobicity directly measures the adhesion ability of LABs to enterocytic cellular lines, which is a most desirable property of probiotic bacteria [19]. Therefore, hydrophobicity was taken as the major criteria for selection of LABs as probiotic and at this stage of examination, out of 20 isolates six showing less than 45 percent hydrophobicity were discontinued. The Lacp28 exhibited best (P<0.05) hydrophobic activity against toluene. The previous report showed the selection of probiotic LAB primarily done on the basis of their superiority (>40 percent) in hydrophobicity against xylene [29] and hexadecane [6] for swine isolates. For efficient probiotic activity, the LAB must have ability to adhere and colonize in intestinal epithelium cells of the host [52]. Adhesion of LAB to intestinal epithelium inhibits colonization of pathogens and retains normal gut mucosal immunity [6]. The surface adhesion was further confirmed through heavy *in vitro* adhesion of Lacp28 to pig intestinal epithelium cells without showing any adhesion towards chicken intestinal epithelium cells (400X magnification), that indicating the host-species specific adhesion of LABs as described earlier also [53]. Similarly, *L*. *acidophilus* PF01 and *L*. *acidophilus* CF07 isolated from piglets and chicken showed heavy *in-vitro* adhesion to duodenal epithelium cells of pig and chicken, respectively [46].

The isolates with extracellular enzyme activity might be helpful in nutrient digestibility by producing synergistic effect [54] providing additional beneficial characteristic to a probiotic. The phytase and protease activities was consistently highest for Lacp28 isolate as compared to other isolates. Kim et al. [31] also reported that LABs isolated from swine intestinal tract displayed different enzyme (amylase, protease, lipase and phytase) activities. In contrary, LABs isolated from poultry were negative for lipase with variable amylase and protease activities [54]. This might be one of the reasons behind the development of species-specific probiotics.

Resistance to antimicrobial substance is a very important selection property for probiotic, as antimicrobial resistant strains can be co-administered with antimicrobial compound for treatment of diseases [54]. All isolates were susceptible to penicillin, azithromycin, clindamycin, erythromycin, cephalothin and chloramphenicol but resistant to ciprofloxacin, ofloxacin, gatifloxacin, vancomycin and co-trimoxazole. Similarly, Shazali et al. [55] reported that LAB isolated from broiler feces sensitive to penicillin, amoxicillin, chloramphenicol and ampicillin, and resistant to ciprofloxacin, gentamycin, kanamycin, streptomycin, vancomycin, bacitracin and tetracycline. In contrast, the LABs isolated from swine feces were resistant to penicillin G, oxytetracycline, lincomycine and susceptible to bacitracin and riframpicin [54]. De Angelis et al. [5] reported that the swine origin *Lactobacilli sp*. (n = 35) were resistant to ampicillin, vancomycin, chloramphenicol, streptomycin, neomycin, nalidixic acid, gentamycin, kanamycin and novobiocin but tetracycline was effective for all the isolates. The above discussion clearly indicates that LAB exhibits highly variable sensitivity towards different antimicrobials, therefore, every probiotic has specific choice of antimicrobial to make a combination.

Antagonistic activity against pathogens is a prerequisite of a potential probiotic. Among the most commonly encountered pathogens are *E*. *coli* and *S*. Typhimurium which are mainly responsible for neonatal diarrhoea in piglets [56]. All selected isolates (n = 14) showed antagonistic activity against nine different pathogens, where Lacp28 displayed highest (P<0.05) antagonistic activity against *E*. *coli*, *S*. Typhimurium, *Staph*. *intermedius*, *Staph*. *chromogenes*, *Proteus mirabillis* and *Areomonas veonii*. Antagonistic activity of LABs might be possibly due to production of antimicrobial substances like organic (lactic and acetic) acids, hydrogen peroxide, bacteriocin, antimicrobial peptides etc. [57]. Highest (P<0.05) antagonism activity was shown by swine isolate, *L*. *reuteri* BFE 1058 against *Enterococcus* sp. and *Listeria monocytogenes* [27], and *L*. *plantarum* against *E*. *coli*, *Staph*. *aureus*, *Salmonella sp*., *Shigella sp*., and *Kiebsiella sp*. [6].

There was a lot of variation among the isolates in their ability to compete with enteric pathogens as the rate of inhibition ranged from 52.0 to 90.48. Similarly, 87% and 77% inhibition in growth of *Staph*. *arueus* and standard *Staph*. *arueus* ATCC25923 was observed when co-cultured with *L*. *plantarum* [35]. In contrast, Venkatesan et al. [57] revealed that among three probiotic (*Lactobacillus* sp., *Bifidobacterium* sp. and *Saccharomyces cerevisiae*) strains *Bifidobacterium* sp. showed highest (P<0.05) co-culture activity with *Escherichia* sp., *Salmonella* sp., *Pseudomonas* sp., *Klebsiella* sp., *Staphylococcus* sp., *Shigella* sp., *Proteus* sp. and *Streptococcus* sp. The suppression of pathogen growth might be possibly due to production of antimicrobial substances like organic (lactic and acetic) acids, hydrogen peroxide, bacteriocin, antimicrobial peptides etc. [58]. Disparity in the antagonism activity against different pathogens indicates that probiotic strains are highly pathogen specific and prerequisite for probiotic potential.

Phylogenetic analysis was done for molecular identification of the best performed isolate Lacp28 by using three sets of primers to have more authenticated in identification. By using universal bacteria primer the isolate could be identified only up to genus level. However, amplification of 16S rDNA with other two primers the isolate Lacp28 was identified as *Pediococcus acidilactici* strain FT28 with 100% similarity. FAO/WHO [3] also recommended that amplification and sequencing of 16S rRNA or rDNA for identification of probiotic strains. Previous report also observed that phylogenetic estimation of 16S rRNA the *P*. *acidilactici* Kp10 was identified from milk product with antagonistic activity and used as potential probiotic [59].

*In vivo* evaluation on early weaned pigs showed a positive impact on FCR with dietary supplementation of *P*. *acidilactici* FT28 and *L*. *acidophilus*, though the gain in weight and daily DM intake did not show any significant variation. Similar finding were reported by Wang et al. [16] that feeding of *L*. *plantarum* ZLP001, isolated from GIT of weaning piglet improved FCR as compared to those fed on diet supplemented with antibiotics.

The supplementation of probiotic, *P*. *acidilactici* FT28 (swine-origin) increased digestibility of CP and CF but not in *L*. *acidophilus* (conventional dairy-origin) fed groups indicating that a culture from the host animal is more effective probiotic as compared to the culture from other sources. Earlier report also showed higher digestibility of DM and CP in weaning piglets [60] and grower-finisher pigs [61] by supplementation of *L*. *salivarius*, *L*. *reuteri* and *L*. *plantarum*. As a natural inhabitants of the GIT of piglets, LAB produce metabolites like lactic acid and digestive enzymes which stimulate peristaltic movements and promote apparent nutrient digestibility [9]. In contrast to our study, higher digestibility of all nutrients was observed in pigs fed different probiotics like Bacillus and *L*. *acidophilus* [62].

The measurement of blood haemato-biochemical profile in farm animals can provide a significant evidence about the health and metabolism of the animals [63]. In the present study, RBC and WBC count were greater (P<0.05) in *P*. *acidilactici* FT28 fed animals compared *L*. *acidophilus* fed group in day 45. However, supplemented groups showed lower (P<0.05) RBC count in day 90. Previous studies also showed a positive correlation between dietary levels of probiotics and haematological indices like RBC and WBC in broiler chickens [19]. In contrary, Prieto et al. [64] revealed that pigs on *B*. *pumilus* treatment had lower (P<0.05) differential leucocytes count than control animals. The concentration Hb was significantly (P<0.001) decreased in both probiotics groups compared to control. In the same line, Hossein et al. [65] showed that feeding of *B*. *subtilis* and *B*. *licheniformis* (Bioplus 2B) @ 0.5 or 1 g/kg of feed in growing lambs resulted a significant (P<0.05) decrease in the level of Hb, increase in mean corpuscular volume (MCV) and mean corpuscular hemoglobin (MCH) levels.

A significant effect was observed for serum glucose level which reflected increasing trend with probiotics of dairy origin to host origin. Probiotic supplementation could alter gut microflora, which may be responsible for modification of gut hormone secretion and improvement of glucose homeostasis [66]. Similar to our results, previous studies also reported with increased (P<0.05) serum glucose level in pigs by supplementation of *Saccharomyces cerevisiae* [67]. The plasma protein concentration at any given time would reflect the function of hormonal balance, nutritional status, water balance and other factors affecting the state of health. A positive effect (P<0.001) was obtained on serum total protein, albumin and globulin content in *P*. *acidilactici* FT28 supplemented group as compared to *L*. *acidophilus* and control, confirming species-speficity of probiotics. Dong et al. [20] also observed that dietary supplementation of *L*. *plantarum* GF103 and *B*. *subtilis* B27 in weaned piglets, increased (P<0.05) serum concentrations of total protein, albumin and globulin creatinine as compared to control animals in post 14 days of feeding. The serum triglyceride level did not show any effect among the treatment groups. In contrast, our previous study reported lower serum triglyceride level by supplementing *P*. *acidilactici* and *L*. *acidophilus* in grower-finisher pigs [14, 22].

Various stresses encountered during weaning process leading to production of reactive oxygen species (ROS), which may oxidize host biomolecules like lipids, proteins, DNA or carbohydrates resulting in dysbalance of functional antioxidative network of the host. However, supplementation of probiotics irrespective of host improved GSH level in packed RBC and catalase in erythrocytes. In the same line, Cai et al. [10] observed that suckling piglets administered with *L*. *fermentum* increased (P<0.05) plasma concentration of glutathione peroxidase activity. The erythrocytic SOD activity was improved by feeding *P*. *acidilactici* FT28 (swine-origin) compared to groups fed either basal feed alone or *L*. *acidophilus*. The present finding was in agreement with Wang et al. [9] who reported that supplementation of swine origin *L*. *plantarum* ZLP001 in weaning piglets increased (P<0.05) serum concentration of superoxide dismutase, glutathione peroxidase and catalase. The present findings suggests that supplementation of swine origin *P*. *acidilactici* FT28 was effective in improving antioxidant status to combat weaning stresses in piglets, when compared with *L*. *acidophilus* (dairy origin).

## Conclusions

The results of this study concluded with a preliminary depiction to establish a species-specific probiotic by applying valid criteria for the selection of isolates. A total of 30 LABs were isolated from the feces of piglets. The isolate Lacp28 identified as *Pediococcus acidilactici* FT28 showed consistent superiority over other isolates. The isolate Lacp28 showed wide spectrum inhibitory activities against number of pathogens including *E*. *coli* and Salmonella. The isolate also possessed other desirable probiotic characteristics like resistance to low *p*H, bile salts, high hydrophobicity and digestive enzyme activities. It also had the capabilities for improvement of feed conversion ratio and blood hematological profile with better species-specificity in protein metabolism and antioxidant profile. But more feeding trials need to be conducted with *P*. *acidilactici* FT28 in pigs to assess dose per kg of weight and frequency of feeding with highest advantages.

## Supporting information

S1 Fig Agarose gel (1.5%) electrophoresis of 16S rRNA gene amplification products of isolate Lacp28 a) 27F/1492R primer, b) Lpig-F/Lpig-R and S-17F/ A-17R primer Lane M and M1: 100 bp plus DNA ladder; Lane M2: 100 bp ladder; Lane 1–3: Lacp28.
(JPG)

S1 Table Carbohydrate utilization† of LAB isolates using HiCarboTM identification system. (DOCX)

## References

[1] Smith AL, Stalder KJ, Serenius TV, Baas TI, Mabry JW. Effect of weaning age on nursery pig and sow reproductive performance. J. Swine Health Prod. 2008; 16, 131–137.

[2] Jin LZ, Ho YW, Abdullah N, Jalaludin S. Growth performance, intestinal microbial populations and serum cholesterol of broilers fed diets containing Lactobacillus cultures. Poult. Sci. 1998; 77, 1259–1265. doi: 10.1093/ps/77.9.1259 9733111 10.1093/ps/77.9.1259

[3] FAO/WHO. Guidelines for the evaluation of probiotics in food Report of a Joint FAO/WHO Working Group on Drafting Guidelines for the Evaluation of Probiotics in Food Ontario Canada April 30 and May 1 2002; pp.1–11.

[4] Singh BR. Axone, a probiotic for pigs promotes growth rate in grower and suckling piglets of large black breed. 2013; https://www.notoare.com/index.php/11591615

[5] De Angelis, Siragusa M, Berloco S, Caputo M, Settanni L, Alfonsi L, et al Selection of potential probiotic lactobacilli from pig feces to be used as additives in pelleted feeding. Res. Microbiol. 2006; 157, 792–801. doi: 10.1016/j.resmic.2006.05.003 16844349 10.1016/j.resmic.2006.05.003

[6] Pringsulaka O, Rueangyotchanthana K, Suwannasai N, Watanapokasin N, Amnueysit P, Sunthornthummas S, et al *In vitro* screening of lactic acid bacteria for multi-strain probiotics. Livest. Sci. 2015; 174, 66–73.

[7] Balasingham K, Valli C, Radhakrishnan L, Balasuramanyam D. Probiotic characterization of lactic acid bacteria isolated from swine intestine. Vet. World 2017; 10(7), 825–829. doi: 10.14202/vetworld.2017.825-829 28831230 10.14202/vetworld.2017.825-829 PMC5553155

[8] Chiang ML, Chen HC, Chen KN, Lin YC, Lin YT, Chen MJ. Optimizing production of two potential probiotic *Lactobacilli* strains isolated from piglet feces as feed additives for weaned piglets. Asian Australas. J. Anim. Sci. 2015; 8(8), 1163–1170. 10.5713/ajas.14.0780 PMC4478485 26104525

[9] Wang AN, Yi XW, Yu HF, Dong B, Qiao SY. Free radical scavenging activity of Lactobacillus fermentum in vitro and its anti-oxidative effect on growing–finishing pigs. J. Appl. Microbiol. 2009; 107, 1140–1148. doi: 10.1111/j.1365-2672.2009.04294.x 19486423 10.1111/j.1365-2672.2009.04294.x

[10] Singh K, Kallali B, Kumar A, Thaker V. Probiotics: a review. Asian Pac. J. Trop. Biomed. 2011; 1, 287–290.

[11] Yadav R, Puniya AK, Shukla P. Probiotic properties of *Lactobacillus plantarum* RYPR1 from an indigenous fermented beverage Raabadi. Frontiers in Microbio. 2016; 7, 1683–1691. 10.3389/fmicb.2016.01683 PMC5073146 27818658

[12] Dowarah R, Verma AK, Agarwal N. The use of Lactobacillus as an alternative of antibiotic growth promoters in pigs: a review. Anim. Nutr. 2017; 3, 1–6. 10.1016/j.aninu.2016.11.002 PMC5941084 29767055

[13] Quilodrán-Vega SR, Villena J, Valdebenito J, Salas MJ, Parra C, Ruiz A, et al Isolation of lactic acid bacteria from swine milk and characterization of potential probiotic strains with antagonistic effects against swine-associated gastrointestinal pathogens. Canadian J. Microbio. 2016; 62(6), 514–524. 10.1139/cjm-2015-0811 27149540

[14] Dowarah R, Verma AK, Agarwal N, Singh P, Patel BHM. Effect of swine based probiotic on performance, diarrhoea scores, intestinal microbiota and gut health of grower-finisher crossbred pigs. Livest. Sci. 2017; 195, 74–79.

[15] Wang J, Ji H, Zhang D, Liu H, Wang S, Shan D, et al Assessment of probiotic properties of *Lactobacillus plantarum* ZLP001 isolated from gastrointestinal tract of weaning pigs. Afr. J. Biotechnol. 2011; 10(54), 11303–11308.

[16] Wang J, Ji HF, Wang SX, Zhang DY, Liu H, Shan DC, et al *Lactobacillus plantarum* ZLP001: *In vitro* Assessment of antioxidant capacity and effect on growth performance and antioxidant status in weaning piglets. Asian Austral. J. Anim. Sci. 2012; 25(8), 1153–1158. 10.5713/ajas.2012.12079 PMC4093004 25049675

[17] Dowarah R, Verma AK, Agarwal N, Singh P. Effect of swine based probiotic on growth performance, nutrient utilization and immune status of early weaned grower-finisher crossbred pigs. Anim. Nutr. Feed Technol. 2016; 16(3), 451–461.

[18] Cai CJ, Cai PP, Hou CL, Zeng XF, Qiao SY. Administration of *Lactobacillus fermentum* I5007 to young piglets improved their health and growth. J. Anim. Feed Sci. 2014; 23, 222–227.

[19] Onifade AA. Growth performance, carcass characteristics, organ measurements and haematology of broiler chickens fed a high fibre diet supplemented with antibiotics or dried yeast. Die Nahrung. 1997; 41: 370–374.

[20] Dong X, Zhang N, Zhou M, Tu Y, Deng K, Diao Q. Effects of dietary probiotics on growth performance, faecal microbiota and serum profiles in weaned piglets. J. Basic Microbiol. 2013; 49(2), 220–226.

[21] Gilliland SE, Nelson CR, Maxwell C. Assimilation of cholesterol by *Lactobacillus acidophilus*. Appl. Environ. Microbiol. 1985; 49, 377–381. 3920964 10.1128/aem.49.2.377-381.1985 PMC238411

[22] Dowarah R, Verma AK, Agarwal N, Singh P. Efficacy of species-specific probiotic *Pediococcus acidilactici* FT28 on blood biochemical profile, carcass traits and physicochemical properties of meat in fattening pigs. Res. Vet. Sci. 2018 (in press accepted). 10.1016/j.rvsc.2017.11.011 29179030

[23] Mishra DK, Verma AK, Agarwal N, Mondal SK, Singh P. Effect of dietary supplementation of probiotics on growth performance, nutrients digestibility and faecal microbiology in weaned piglets. Anim. Nutr. Feed Technol. 2014; 14(2), 283–290.

[24] Tamura K, Peterson D, Peterson N, Stecher G, Nei M, Kumar S. MEGA5: molecular evolutionary genetics analysis using maximum likelihood, evolutionary distance, and maximum parsimony methods. Mol. Biol. Evol. 2011; 28, 2731–2739. doi: 10.1093/molbev/msr121 21546353 10.1093/molbev/msr121 PMC3203626

[25] Lane DJ. 16S/23S rRNA sequencing In Stackebrandt E. and Goodfellow M. (ed.), Nucleic acid techniques in bacterial systematics. John Wiley & Sons, New York, NY p. 115–147; 1991.

[26] Heilig HGHJ Zoetendal EG, Vaughan EE, Marteau P, Akkermans ADL, Willem MV. Molecular diversity of *Lactobacillus spp*. and other lactic acid bacteria in the human intestine as determined by specific amplification of 16S ribosomal DNA. Appl. Environ. Microbiol. 68(1), 2002; 114–123. doi: 10.1128/AEM.68.1.114-123.2002 11772617 10.1128/AEM.68.1.114-123.2002 PMC126540

[27] Du Toit M, Franz PAMC, Dicks TML, Schillinger U, Haberer P, Warlies B. et al Characterization and selection of probiotic lactobacilli for a preliminary minipig feeding trial and their effect on serum cholesterol levels, faeces pH and faeces moisture content. Int. J. Food Microbiol. 1998; 40, 93–104. 9600615 10.1016/s0168-1605(98)00024-5

[28] Gotcheva V, Hristozova E, Hristozova T, Guo M, Roshkova Z, Angelov A. Assessment of potential probiotic properties of lactic acid bacteria and yeast strains. Food Biotechnol. 2002; 16, 211–225.

[29] Palomares IC, Morales PR, Felix AE. Evaluation of probiotic properties in *Lactobacillus* isolated from small intestine of piglets. Revista Latinoamericana de Microbiologia. 2007; 49 (3–4), 46–54.

[30] Vlkova E, Rada V, Popelarova P, Trojanova I, Killer J. Antimicrobial susceptibility of *Bifidobacteria* isolated from gastrointestinal tract of calves. Livest. Sci. 2006; 105(1–3), 253–259.

[31] Kim P, Min J, Chang HY, Kim SJ, Park YH. Probiotic properties of *Lactobacillus* and *Bifidobacterium* strains isolated from porcine gastrointestinal tract. Appl. Environ. Microbiol. 2007; 74, 1103–1111. 10.1007/s00253-006-0741-7 17136367

[32] Schillinger U, Lucke KF. Antibacterial activity of Lactobacillus sake isolated from meat. Appl. Environ. Microbiol. 1989; 55(8), 1901–1906. 2782870 10.1128/aem.55.8.1901-1906.1989 PMC202976

[33] Soleimani NA, Kermanshahi RK, Yakhchali B, Nejad TS. Antagonistic activity of probiotic lactobacilli against *Staphylococcus aureus* isolated from bovine mastitis. Afr. J. Microbiol. Res. 2010; 4(20), 2169–2173.

[34] Lim S, Dong-Soon IM. Screening and characterization of probiotic lactic acid bacteria isolated from Korean fermented foods. J. Microbiol. Biotechnol. 2009; 19(2), 178–186. 19307768 10.4014/jmb.0804.269

[35] Kos B, Uskovic JS, Vukovic S, Impraga MS, Frece J, Matosic S. Adhesion and aggregation ability of probiotic strain *Lactobacillus acidophilus* M92. J. Appl. Microbiol. 2003; 94, 981–987. 12752805 10.1046/j.1365-2672.2003.01915.x

[36] NRC. *Nutrient Requirements of Swine*. 10th Revised edition. National Academy Press, Washington, DC; 1998.

[37] Agarwal N, Kamra DN, Chaudhary LC, Agarwal I, Sahoo A, Pathak NN. Microbial status and enzyme profile of crossbred calves fed on different microbial feed additives. Lett. Appl. Microbiol. 2002; 34, 329–336. 11967054 10.1046/j.1472-765x.2002.01092.x

[38] AOAC. Association of official analytical chemists (19th ed.). Gaithersburgh, Maryland, USA: Association of Official Methods of Analysis; 2000.

[39] Marklund S, Marklund G. Involvement of the superoxide anion radical in the autoxidation of pyrogallol and a convenient assay for superoxide dismutase. Eur. J. Biochem. 1974; 47, 469–474. 4215654 10.1111/j.1432-1033.1974.tb03714.x

[40] Menami M, Yoshikawa H. A simplified assay method of superoxide dismutase activity for clinical use. Clin. Chim. Acta. 1979; 92(3), 337–342. 436274 10.1016/0009-8981(79)90211-0

[41] Bergmeyer HU. In: Methods of Enzymatic Analysis. Vol. III, Weinhein, Deer field Beach, Florida, 1983; Pp. 273.

[42] Prins HK, Loos JA. Biochemical methods in red cell genetics. Academic Press, London, 1969; Pp, 127–129.

[43] Choubey M, Pattanaik AK, Baliyan S, Dutta N, Jadhav SE, Sharma K. Dietary supplementation of a novel phytogenic feed additive: effects on nutrient metabolism, antioxidant status and immune response of goats. Anim. Prod. Sci. 2015; doi: 10.1071/AN14770

[44] Duncan DB. Multiple range and multiple F-test. Biometrics 1995; II, 1–42.

[45] Jassim RAMA. *Lactobacillus ruminis* is a predominant lactic acid producing bacterium in the caecum and rectum of the pig. Lett. Appl. Microbiol. 2003; 37(3), 213–217. 12904222 10.1046/j.1472-765x.2003.01380.x

[46] Ahn YT, Lim KL, Ryu JC, Kang DK, Ham JS, Jang YH et al Characterization of *Lactobacillus acidophilus* isolated from Piglets and Chicken. Asian-Aust. J. Anim. Sci. 2002; 15(12), 1790–1797.

[47] Du Toit M, Dicks LMT, Holzapfe WH. Identification of hetero-fermentative lactobacilli isolated from pig faeces by numerical analysis of total soluble cell protein patterns and RAPD-PCR. Lett. Appl. Microbiol. 2003; 37, 12–16. 12803548 10.1046/j.1472-765x.2003.01334.x

[48] Ashmaig A, Hasan A, Gaali EEL. Identification of lactic acid bacteria isolated from traditional Sudanese fermented camel’s milk (Gariss). Afr. J. Microbiol. Res. 2009; 3(8), 451–457.

[49] Saarela M, Mogensen G, Fondén R, Matto J, Sandholm TM. Probiotic bacteria: safety, functional and technological properties. J. Biotech. 2000; 84, 197–215. 10.1016/s0168-1656(00)00375-8 11164262

[50] Ouwehand AC, Kirjavainen PV, Shortt C, Salminen S. Probiotics: mechanisms and established effects. Int. Dairy J. 1999; 9, 43–52.

[51] Guerra NP, Bernardez PF, Mensez J, Cachaldora P, Castro LP. Production of four potentially probiotic lactic acid bacteria and their evaluation as feed additives for weaned piglets. Anim. Feed Sci. Technol. 2007; 134, 89–107.

[52] Barrow PA, Brooker BE, Fuller R, Newport MJ. The attachment of bacteria to the gastric epithelium of the pig and its importance in the micro-ecology of the intestine. J. Appl. Bacteriol. 1980; 48,147–154. 6246061 10.1111/j.1365-2672.1980.tb05216.x

[53] Taheri HR, Moravej H, Tabandeh F, Zaghari M, Shivazad M. Screening of lactic acid bacteria toward their selection as a source of chicken probiotic. Poult. Sci. 2009; 88, 1586–1593. doi: 10.3382/ps.2009-00041 19590072 10.3382/ps.2009-00041

[54] Petsuriyawong B, Khunajakr N. Screening of probiotic lactic acid bacteria from piglet feces. Kasetsart J (Nat. Sci.) 2011; 45, 245–253.

[55] Shazali N, Foo HL, Loh TC, Choe DW, Rahim RA. Prevalence of antibiotic resistance in lactic acid bacteria isolated from the faeces of broiler chicken in Malaysia. Gut Pathogens 2014; 6, 1–7. doi: 10.1186/1757-4749-6-1 24447766 10.1186/1757-4749-6-1 PMC3902413

[56] Fairbrother JM, Nadeau E, Gyles CL. *Escherichia coli* in post weaning diarrhea in pigs: an update on bacterial types, pathogenesis, and prevention strategies. Anim. Health Res. Rev. 2005; 6, 17–39. 16164007 10.1079/ahr2005105

[57] Venkatesan S, Kirithika M, Roselin I, Ganesan R, Muthuchelian K. Comparative *in vitro* and *in vivo* study of three probiotic organisms, *Bifidobacterium* sp., *Lactobacillus* sp., *S*. *Cerevisiae* and analyzing its improvement with the supplementation of prebiotics. Int. J. Pl. An. Env.Sci. 2012; 2(2), 94–106.

[58] Cho IJ, Lee NK, Hahm YT. Characterization of *Lactobacillus spp*. isolated from the feces of breast-feeding piglets. J. Biosci. Bioeng. 2009; 108(3), 194–198. doi: 10.1016/j.jbiosc.2009.03.015 19664551 10.1016/j.jbiosc.2009.03.015

[59] Abbasiliasi S, Tan JS, Ibrahim TAT, Ramanan RN, Vakhshiteh F, Mustafa S, et al Isolation of *Pediococcus acidilactici*Kp10 with ability to secrete bacteriocin-like inhibitory substance from milk products for applications in food industry. BMC Microbiol. 2012; doi: 10.1186/1471-2180-12-260 23153191 10.1186/1471-2180-12-260 PMC3571982

[60] Zhao PY, Kim IH. Effect of direct-fed microbial on growth performance, nutrient digestibility, fecal noxious gas emission, fecal microbial flora and diarrhea score in weanling pigs. Anim. Feed Sci. Technol. 2015; 200: 86–92.

[61] Shon SK, Hong JW, Min BJ, Lee WB, Kim IH. Effects of *Lactobacillus reuteri*-based direct-fed microbial supplementation for growing-finishing pigs. Asian Austral. J. Anim. Sci. 2005; 18: 370–374.

[62] Balasubramanian B, Lee SI, Kim IH. Inclusion of dietary multi-species probiotic on growth performance, nutrient digestibility, meat quality traits, faecal microbiota and diarrhoea score in growing–finishing pigs. Italian J. Anim. Sci. 2017; 1–7.

[63] Friendship RM, Henry HC. Cardiovascular System, Hematology, and Clinical Chemistry In: Diseases of Swine, Leman AD., Straw B.E., Mengeling W.L., D'Allaire S. and Taylor D.J. (Eds.). Iowa State University Press, Ames, LA 1992; pp: 3–10.

[64] Prieto ML, Laurie OS, Shiau PT, McLoughlin P, O’Donovan O, Rea MC, et al Evaluation of the efficacy and safety of a marine-derived bacillus strain for use as an in-feed probiotic for newly weaned pigs. PLoS ONE 2014; 9(2): e88599 doi: 10.1371/journal.pone.0088599 24586349 10.1371/journal.pone.0088599 PMC3935854

[65] Hossein AA, Alireza ME, Mohammad R, Majid M. Effects of *Bacillus subtilis* and *Bacillus licheniformis*-based probiotic on performance, hematological parameters and blood metabolites in lambs. Intl. J. Food Nutr. Sci. 2014; 3(4): 8–15.

[66] Yadav H, Jain S, Sinha PR. Oral administration of dahi containing probiotic *Lactobacillus acidophilus* and *Lactobacillus casei* delayed the progression of streptozotocin-induced diabetes in rats. J. Dairy Res. 2008; 75(2), 189–195. doi: 10.1017/S0022029908003129 18474136 10.1017/S0022029908003129

[67] Kumar S, Verma AK, Mondal SK, Gupta M, Patil A, Jangi BL. Effect of live *Saccharomyces cerevisiae* feeding on serum biochemistry in early weaned cross bred piglets. Vet. World 2012; 5(11), 663–666.

