# Supplementary figures and images for: Selection and characterization of probiotic lactic acid bacteria and its impact on growth, nutrient digestibility, health and antioxidant status in weaned piglets
Source: PLoS One. 2018 Mar 8;13(3):e0192978. doi: 10.1371/journal.pone.0192978 (PMC5843174; doi:10.1371/journal.pone.0192978)

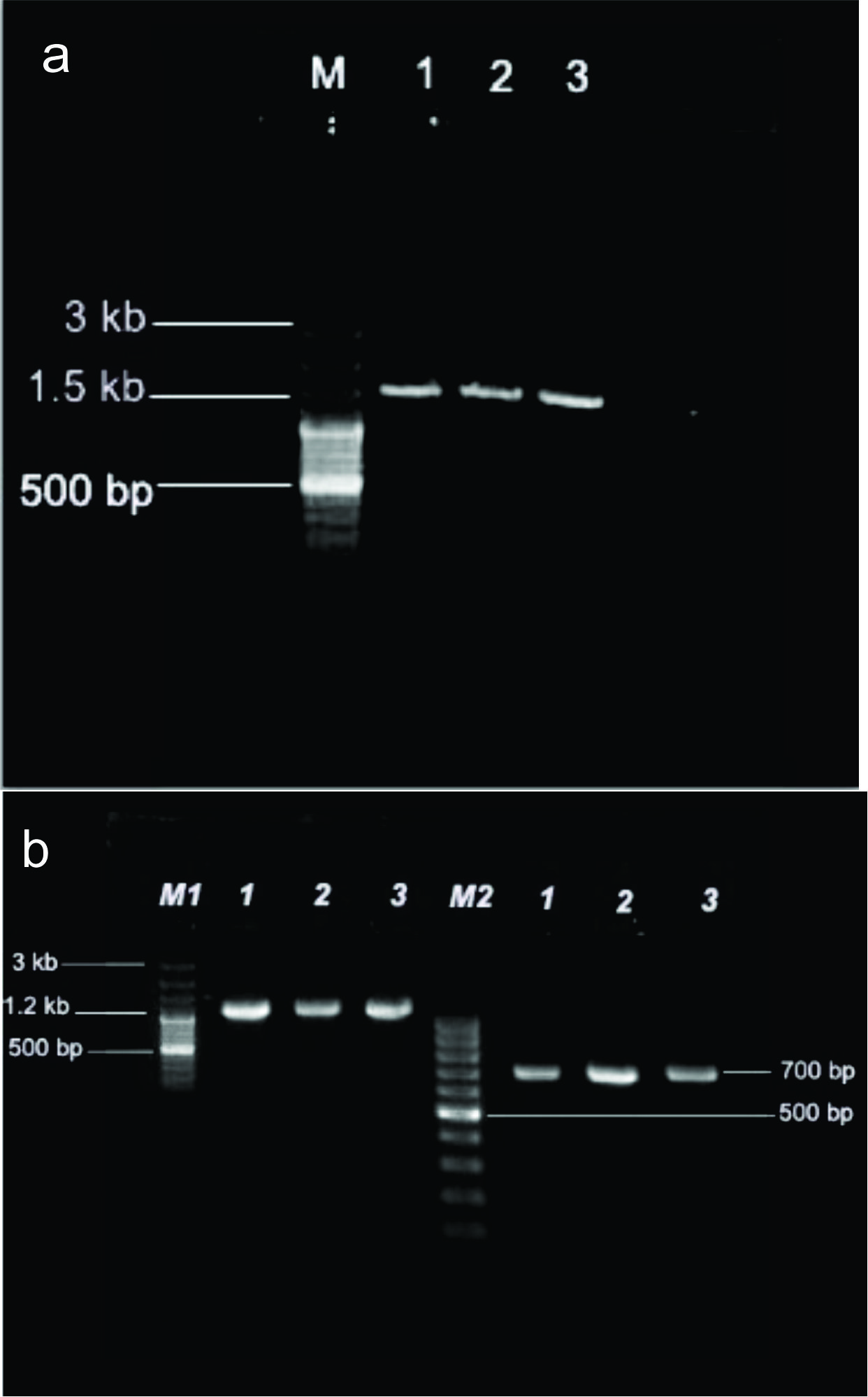

Supplement: S1 Fig — Agarose gel (1.5%) electrophoresis of 16S rRNA gene amplification products of isolate Lacp28 a) 27F/1492R primer, b) Lpig-F/Lpig-R and S-17F/ A-17R primer Lane M and M1: 100 bp plus DNA ladder; Lane M2: 100 bp ladder; Lane 1–3: Lacp28. (JPG) [file pone.0192978.s001.jpg]
